# A Comparison of Midline and Tracheal Gene Regulation during *Drosophila* Development

**DOI:** 10.1371/journal.pone.0085518

**Published:** 2014-01-20

**Authors:** Sarah K. R. Long, Eric Fulkerson, Rebecca Breese, Giovanna Hernandez, Cara Davis, Mark A. Melton, Rachana R. Chandran, Napoleon Butler, Lan Jiang, Patricia Estes

**Affiliations:** 1 Department of Biological Sciences, North Carolina State University, Raleigh, North Carolina, United States of America; 2 Department of Biological and Physical Sciences, Saint Augustine’s University, Raleigh, North Carolina, United States of America; 3 Department of Biological Sciences, Oakland University, Rochester, Michigan, United States of America; Vlaams Instituut voor Biotechnologie and Katholieke Universiteit Leuven, Belgium

## Abstract

Within the *Drosophila* embryo, two related bHLH-PAS proteins, Single-minded and Trachealess, control development of the central nervous system midline and the trachea, respectively. These two proteins are bHLH-PAS transcription factors and independently form heterodimers with another bHLH-PAS protein, Tango. During early embryogenesis, expression of Single-minded is restricted to the midline and Trachealess to the trachea and salivary glands, whereas Tango is ubiquitously expressed. Both Single-minded/Tango and Trachealess/Tango heterodimers bind to the same DNA sequence, called the CNS midline element (CME) within *cis*-regulatory sequences of downstream target genes. While Single-minded/Tango and Trachealess/Tango activate some of the same genes in their respective tissues during embryogenesis, they also activate a number of different genes restricted to only certain tissues. The goal of this research is to understand how these two related heterodimers bind different enhancers to activate different genes, thereby regulating the development of functionally diverse tissues. Existing data indicates that Single-minded and Trachealess may bind to different co-factors restricted to various tissues, causing them to interact with the CME only within certain sequence contexts. This would lead to the activation of different target genes in different cell types. To understand how the context surrounding the CME is recognized by different bHLH-PAS heterodimers and their co-factors, we identified and analyzed novel enhancers that drive midline and/or tracheal expression and compared them to previously characterized enhancers. In addition, we tested expression of synthetic reporter genes containing the CME flanked by different sequences. Taken together, these experiments identify elements overrepresented within midline and tracheal enhancers and suggest that sequences immediately surrounding a CME help dictate whether a gene is expressed in the midline or trachea.

## Introduction

The genes expressed within a particular cell type control its developmental fate and physiological potential. Early in development, master control genes play pivotal roles in controlling cell fate and most master control genes are transcription factors that promote their own expression as well as a variety of downstream target genes. Each target gene, in turn, contributes to tissue development by regulating cellular processes, such as 1) morphology 2) interactions with surrounding cells through signaling, 3) cell divisions and/or 4) the expression of additional genes. To understand how genes are differentially regulated within tissues, we compare the development and gene expression of two tissues in the *Drosophila* embryo: the central nervous system (CNS) midline and the trachea. In *Drosophila*, Single-minded (Sim) is the master control gene of CNS midline cells [1]–[3], while Trachealess (Trh) plays a large role in the development of the fly’s respiratory system, the trachea [4]–[6]. Both Sim and Trh are bHLH-PAS proteins and independently heterodimerize with a common partner, Tango (Tgo), before binding to DNA and activating transcription [7], [8]. Tgo is also a bHLH-PAS protein and paradoxically, Sim/Tgo and Trh/Tgo both bind to a shared five base pair recognition sequence, ACGTG, called the CNS midline enhancer element (CME). Tgo is ubiquitously expressed, whereas Sim is restricted to the midline [9] and Trh to the trachea and a few other tissues, including the salivary gland, filzkorper and CNS [4], [5]. In most cells, Tgo is located in the cytoplasm, but within cells that express one of its partner proteins, such as Sim or Trh, Tgo is transported to the nucleus and upregulated [10]. Once in the nucleus, Sim/Tgo and Trh/Tgo activate overlapping and distinct gene sets [7], [8], [11]–[13].

### Single-minded and the Midline

**Figure 1:**
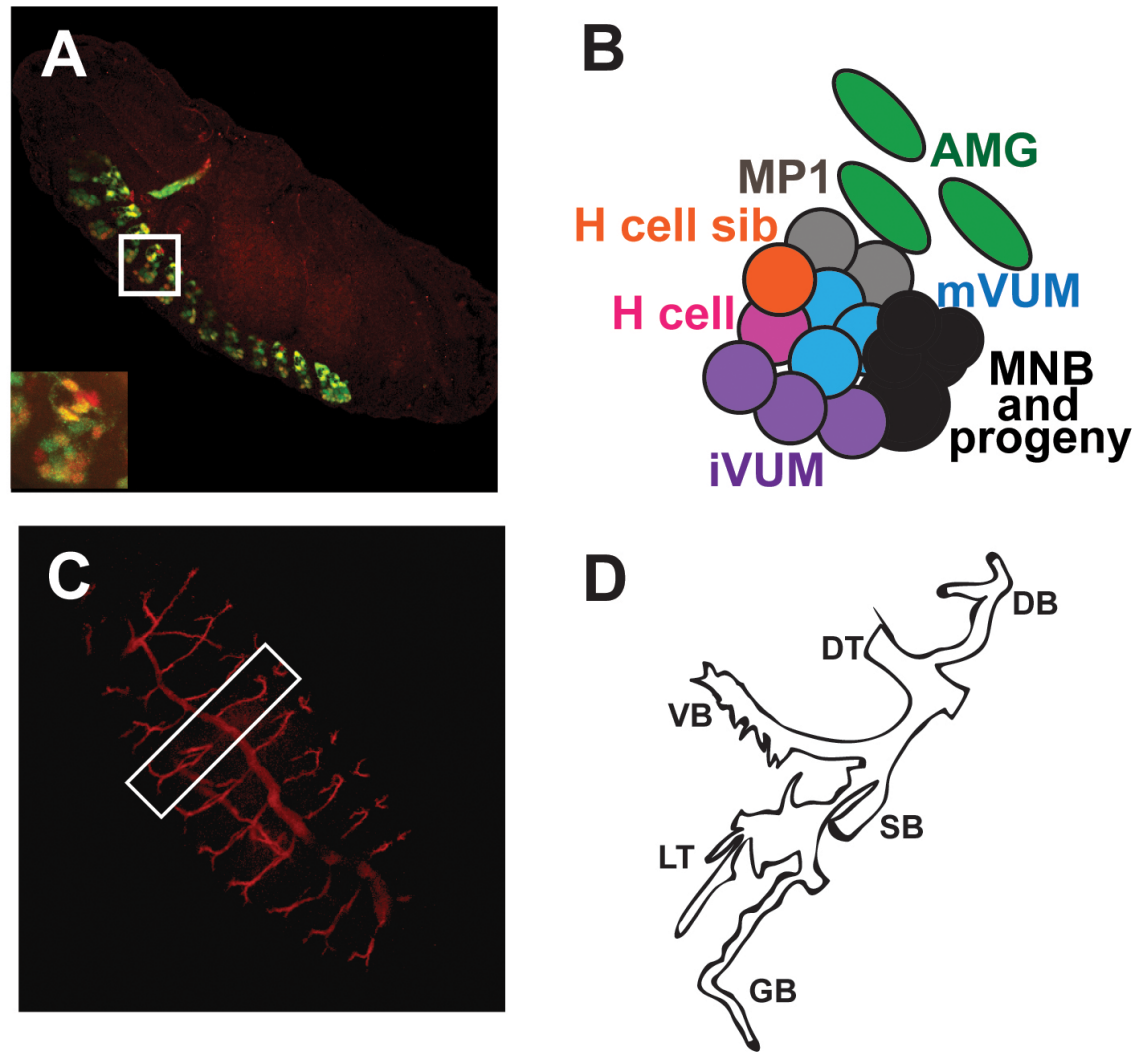
Relative locations of the CNS midline and trachea within the late *Drosophila* embyo. (A) The midline cellular pattern is segmentally repeated throughout the ventral nerve cord at embryonic stage 16. (B) Each segment consists of six neural subtypes and three surviving midline glia whose relative locations within a typical thoracic segment (white box and inset in A) are shown. The midline subtypes include: the MP1 neurons (gray), the H cell (pink), the H cell sib (orange), the ventral unpaired interneurons (iVUMs; purple), the ventral unpaired motorneurons (mVUMs; blue), median neuroblast (MNB) and its progeny (black) and the anterior midline glia (AMG; green); adapted from [24], [108]. (C) By the end of embryogenesis, the trachea form an extensive network that mediates gas exchange throughout the organism. (D) Each tracheal metamere consists of the major dorsal trunk (DT), a dorsal branch (DB), and the visceral (VB), spiracular (SB) and ganglionic (GB) branches and lateral trunk (LT) on the ventral side; adapted from [71]. Lateral views of whole mount embryos stained with anti-*GFP* (green), anti-*sim* (red; A) antibodies or monoclonal antibody *2A12* (red; C) and analyzed by confocal microscopy are shown. (A) The embryo contains a reporter gene that expresses *GFP* in all midline cells.

The embryonic midline and trachea differ in many ways and the following is a brief summary and comparison of the development of these tissues during *Drosophila* embryogenesis. CNS midline cells are specified early in embryogenesis when *sim* is activated prior to gastrulation, in a single row of cells sandwiched in between the mesoderm and ectoderm on each side of the embryo; cells called the mesectoderm [9]. Sim protein is first expressed during gastrulation as the two rows of mesectodermal cells come together at the ventral midline. After meeting ventrally, midline cells invaginate to form a signaling center that organizes the CNS as it matures symmetrically on either side of the midline. As CNS axons differentiate, midline glia secrete *Netrin (Net) A* and *B* to attract axons to cross the midline [14]–[16] and then *slit* to prevent recrossing [17]–[19]. Some axons continually express *roundabout (robo),* the receptor for *slit* [18], at the growth cone surface and never cross the midline, whereas axons that cross the midline require *commissureless (comm)* to temporarily prevent *robo* localization at the growth cone, allowing them to cross [20]–[23]. During mid to late embryogenesis, midline cells differentiate into glia and six neural subtypes that can be distinguished based on their gene expression patterns (Fig. 1A–B) [11], [24]. By the time the embryo hatches into a larva, most midline neurons have differentiated and begun to secrete subtype specific neurotransmitters and make connections with target tissues [24], [25]. In addition, the midline glia have enwrapped and secured the CNS axons that cross the midline [1], [26].

### Trachealess and the Trachea

In *Drosophila,* the trachea are a network of air-filled tubes constructed during embryogenesis that function in gas exchange (reviewed in [27]–[30]). Tracheal cells can first be recognized during *Drosophila* gastrulation when *ventral veinless* (*Vvl*) and *trh* are activated by *JAK/STAT* signaling [31]–[34] within segmentally repeated tracheal pits or placodes [5], [35]. *Decapentaplegic* (*Dpp*) and *Epidermal Growth Factor (EGF)* signaling limit the embryonic dorsal and ventral boundaries of the trachea, while *wingless* (*wg)* restricts the location of trachea within each segment [4], [5], [36]. As development progresses, terminal cells at the end of the growing tracheal tubes lead migration into tissues and specialized cells fuse to connect the separate, developing metameric trachea, creating a continuous tubular network. Fusion of lateral and dorsal trunks is facilitated by the Dysfusion (Dys) bHLH-PAS protein, another partner of Tgo [37]–[40] and after fusion, the two major tracheal tubes, called dorsal trunks, span the length of the embryo (Fig. 1C and D). Interestingly, insect trachea share functional and developmental similarities with the vertebrate vasculature. Both are interconnecting and branched tubular networks, function in gas exchange, and are patterned by related developmental genes and mechanisms [41]. For instance, signaling by *fibroblast growth factor (FGF),* called *breathless (btl)* in flies [42], [43], plays a key role in the formation of both of these tissues. Btl is expressed in all tracheal cells and leading cells of nascent branches interact with neighboring tissues through their production of the FGF signal, *branchless,* which stimulates and guides branch formation [44]. FGF signaling, together with the *Drosophila hypoxia inducible factor*, also guides later growth and branching of the trachea, driven, in part, by oxygen demands of tissues [41]. At the end of embryogenesis, the tracheal network fills with air and for the remainder of the fly’s life, the trachea delivers oxygen to its tissues.

### Common and Distinct Genes and their Regulation within the Midline and Trachea

The functions and morphology of midline and tracheal cells differ, yet certain aspects of their embryonic development are similar. Both cell types are derived from the ectoderm (the midline is derived from the more specialized mesectoderm) and project long cellular extensions to form specialized contacts with many different cell types [45]–[48]. Moreover, midline glia and tracheal cells provide vital nutrients, growth factors and oxygen for active neurons within the mature embryo and larvae [6], [26]. While Sim is restricted to the midline and Trh to tracheal cells within the embryo, many genes are expressed in both the midline and trachea, including the Vvl POU domain transcription factor, which is needed to activate genes in both tissues [49], [50]. In addition, many signaling pathways, including *Notch, FGF, EGF, engrailed, wg* and *hedgehog (hh)* [11], [12], [26], [47], [51], [52] provide positional cues to regulate development of various cell types within both tissues. Downstream components of these signaling pathways combine with Sim and Trh in unique ways to regulate different gene sets in the midline and tracheal cells. Differences between the two tissues are likely due to the presence of additional, unknown tissue specific proteins that combine with Sim and Trh in unique ways to control gene expression and alter cell activity. In support of this idea, exchanging the PAS domains between Sim and Trh indicates these domains determine target gene specificity, presumably by binding to co-factors restricted to either the midline or trachea [13]. This is consistent with the known properties of PAS domains, which bind many different molecules and co-factors to respond to the environment [53]–[55]. Such co-factors may cause the Sim/Tgo and Trh/Tgo heterodimers to recognize slightly different DNA binding sites within enhancer regions of target genes. The goal of these experiments is to understand how Sim and Trh bind the same protein partner and DNA sequence, yet activate different gene sets in midline and tracheal cells.

To compare regulatory functions of Sim/Tgo and Trh/Tgo during fly development, we selected genes expressed in the midline, trachea or both tissues, identified enhancers that control the expression of each gene and compared them to previously identified midline and tracheal enhancers. To test the importance of previously identified sequence motifs, we generated synthetic reporters that contain the CME combined with binding sites for other factors expressed in the midline or trachea. To further analyze these enhancer sets, we searched for novel motifs common to both, as well as motifs unique to either midline or tracheal genes. The results identify sequence contexts, both proximal and distal to the CME, which promote midline and/or tracheal expression.

## Materials and Methods

### Production of Midline and Tracheal Reporter Genes and Transgenic Strains

*Drosophila melanogaster* genomic sequences encompassing select genes expressed in the midline and trachea were compared across the 12 sequenced *Drosophila* genomes [56] using the USCS genome browser (genome.ucsc.edu). The sequences examined included all introns within a gene and the intergenic regions located between the midline gene and its neighboring upstream and downstream gene. Identified regions conserved in at least 11 of the 12 genomes were first amplified within fragments ranging from ∼200–3500 bp using the primers listed in Table S1 and genomic DNA isolated from the *yw^67^ Drosophila melanogaster* strain. These fragments were either cloned into the *pSTBlue1* intermediary vector and then into the *pHstinger* vector [57] using XhoI/KpnI digestion, or cloned into *pCR8/GW/TOPO* (Invitrogen) and transferred into *pMintgate* using the Gateway system [58]. Minor changes to this cloning scheme are noted below. Transgenic fly lines were generated with the *pHstinger* constructs using standard procedures and three independent lines analyzed for each *GFP* reporter gene. *pMintgate* constructs were injected into the φC31 genomic destination site attP2 (68A1-B2) as previously described [58].

**Figure 2:**
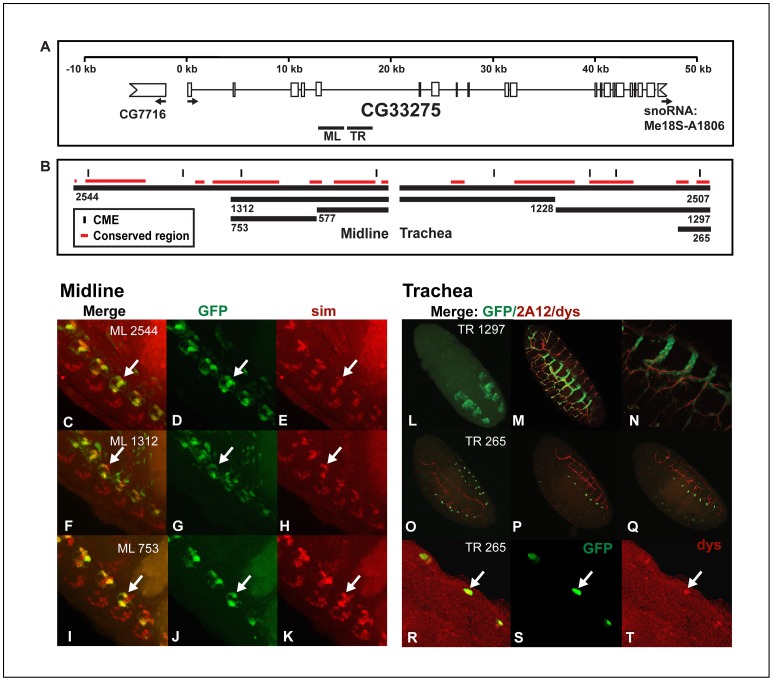
*CG33275* contains a midline enhancer that is separable and distinct from a nearby tracheal enhancer. (A) Locations of regions within the fifth intron of *CG33275* used to generate the reporter constructs are shown. A scale is indicated on top and the thick lines represent the regions analyzed in (B–T). White boxes represent exons and thin lines represent introns. The indented boxes indicate flanking genes, *CG7716* and *snoRNA*, and arrows indicate the start and direction of transcription. (B) Fragments used to generate reporter constructs are shown. The size of each fragment is indicated below it, vertical lines indicate locations of CMEs and red lines represent sequence blocks conserved in at least 11 *Drosophila* species. (C–T) Whole mount embryos were double-stained with anti-*GFP* (green: D, G, J, L and S), anti-*sim* (red; E, H, K), anti-*dys* (red; T) antibodies and monoclonal antibody *2A12* (red; M–Q) and analyzed by confocal microscopy. The overlap in expression is shown in yellow in the merge images (C, F, I, M–Q and R). Reporters (C–E) *CG33275 ML2544:GFP*, (F–H) *CG33275 ML1312:GFP* and (I–K) *CG33275 ML753:GFP* drove expression in midline glia. Midline glia can be identified by the overlap in expression of *GFP* and *sim* (arrows C–K) and are located on the dorsal side of the nerve chord. Midline neurons are located on the ventral side of the nerve chord and labeled by *sim*, but do not express *CG33275* or any of the *CG33275* reporter genes. (L–T) Monoclonal antibody *2A12* labels the tracheal lumen and the anti-*dys* antibody labels tracheal fusion cells. *CG33275 TR1297:GFP* is expressed in all trachea, beginning in the tracheal pits at stage 12 (L) and extending to late embryogenesis (M–N), while *CG33275 TR265:GFP* is expressed only in fusion cells (O–T), indicated by co-localization of *GFP* and Dys (arrows R–T). Lateral views of stage 16 transgenic embryos are shown; anterior is in the top, left hand corner and ventral is on the left, except (L), which is a dorsal view of a stage 12 embryo.

*CG33275.* The *CG33275 ML577* fragment was generated by first digesting the *CG33275 ML 2544*:*GFP* construct in *pSTBlue* with BglII, re-ligating it and then subcloning the remaining 577 bp fragment into *pHstinger*. The *CG33275 ML 1312* fragment was generated from the *CG33275 ML2544:GFP* construct using KpnI/SwaI digestion and blunt end ligation, which removed 1232 bp from the original 2544 bp construct (Fig. 2B). The remaining 1312 bp fragment was then subcloned into *pHstinger*.

*liprin γ*. The *liprin γ* 1781 fragment was generated from the *liprin γ 3141:GFP* construct using SacII/BamHI digestion, as previously reported [59].

### Production of Synthetic Reporter Genes

To generate synthetic reporters, the forward and reverse primer pairs listed in Table S2 were phosphorylated, annealed, ligated and multimers consisting of four copies were separated on 12% polyacrylamide gels, excised and purified with isobutanol extraction. The multimers were first cloned into EcoRI-digested Bluescript KS^−^ and subsequently into *pHstinger* using KpnI/BamHI digestion. Each reporter gene was introduced into the *Drosophila* genome using P element mediated transformation and the *GFP* expression pattern of at least three transgenic lines examined.

### Immunohistochemistry and Confocal Microscopy

Embryos were collected and labeled with antibodies as previously described [60]. The following antibodies were used to localize proteins in *Drosophila* embryos: rat anti-*sim* (1∶100) [10], rabbit anti-*GFP* (1∶500; Molecular Probes, Life Technologies), mouse anti-*GFP* (1∶200; Promega), rabbit anti-*dys* (1∶400) [39]; rabbit anti-*odd-skipped* (*odd;* 1∶400; Jim Skeath, Washington University School of Medicine, St. Louis, MO, USA); and anti-*reversed polarity* (*repo*; 1∶30), anti-*engrailed* (1∶1), and *2A12* (1∶5) MAbs (Developmental Studies Hybridoma Bank). Secondary antibodies were used at 1∶200 and included anti-rat-Alexa568, anti-mouse-Alexa568, anti-rabbit-Alexa405 and anti-rabbit-Alexa488 (Molecular Probes). Images were obtained on a Zeiss 710 in the Cellular and Molecular Imaging Facility at NCSU.

### Motif Identification

MEME [61] was used to identify common motifs in midline and tracheal enhancers. We included previously identified midline enhancers for *sim, Toll, slit* [62], [63], *rhomboid (rho)* [50], *btl* [8], *Vvl* [64], *roughest (rst)* [65], [66], *wrapper* [67], *gliolectin, organic anion transporter protein26f, liprin γ* [59] and *link* [68], as well as the new midline enhancers reported here: *CG33275, NetB, comm, escargot (esg)* and *Ectoderm3 (Ect3)*. The following previously identified tracheal enhancers were included: *trh, Vvl early* [34], *Vvl autoregulatory* [64], *CG13196, CG15252*, *dys* [58], *btl* [8], *rho* [50], *link* [68], and the new tracheal enhancers described here: *CG33275, NetB, liprin γ, esg* and *moody*.

## Results

To understand how diverse genes are transcriptionally regulated in the midline, trachea or both tissues, we identified and compared enhancers of seven genes that are expressed during *Drosophila* development. The seven genes studied include three genes that encode axon guidance and synaptic proteins: *liprin γ, comm and Net B;* a gene in the *EGFR* signaling pathway, *CG33275*; a G protein coupled receptor, *moody*; a cell death gene, *Ectoderm 3 (Ect3)*, and finally, the *esg* transcription factors. Several of these contain large introns and are separated from other genes by large intergenic regions and, therefore, to facilitate the identification of midline and tracheal enhancers, we searched for sequences conserved in a relatively large number of the sequenced *Drosophila* species [56]. We tested the ability of the conserved regions to drive expression in midline and tracheal cells by fusing them to *GFP* within the *pHstinger* or *Mintgate* enhancer tester vectors and generating transgenic fly lines. In certain cases, we also identified a minimal region capable of driving tissue specific expression. The composition and expression patterns of the identified enhancers are briefly summarized below.

### CG33275

This gene is a guanyl-nucleotide exchange factor expressed in both the midline and trachea during embryogenesis [69], [70]. The entire gene spans approximately 47 kb and consists mostly of large introns (Fig. 2A). We identified an enhancer within the fifth intron of *CG33275* capable of driving high levels of *GFP* in midline glia and a separate and distinct tracheal enhancer downstream of the midline enhancer (Fig. 2A and B). The midline enhancer was identified by testing reporter genes *CG33275 ML2544:GFP, ML1312:GFP, ML753:GFP* and *ML577:GFP,* and all but the *CG33275 ML577:GFP* reporter drove expression in midline glia (Fig. 2C–K), in a pattern similar to that of the endogenous gene [11]. The *CG33275 ML753:GFP* midline glial enhancer contains two regions conserved in 12 *Drosophila* species and one of these contains a CME (Fig. 2B). Sequences located just downstream of the midline enhancer drove high levels of *GFP* expression in a pattern similar to the endogenous gene [70]; in all tracheal cells beginning at stage 11 (Fig. 2L–N) and throughout larval stages (not shown). Both tracheal reporter genes *CG33275 TRH2507:GFP* (not shown) and the smaller *CG33275 TRH1297:GFP* reporter gene drove the same tracheal expression pattern (Fig. 2L–N). The *CG33275 TRH1228:GFP* reporter was not expressed in trachea or midline cells (not shown), whereas the *CG33275 TRH265:GFP* reporter was restricted to tracheal fusion cells (Fig. 2O–T), as demonstrated by the overlap in expression with *dys* (Fig. 2R–T). The *CG33275 TRH2507:GFP* reporter contains four CMEs, *CG33275 TRH1297:GFP* contains three of these and *CG33275 TRH265:GFP* contains one. All three of these reporters contain a region with a CME that is conserved across 12 *Drosophila* species (Fig. 2B). Dys, related to Trh, also heterodimerizes with Tgo and binds a site related to the CME, TCGTG, and can weakly interact with the sequences, TCGTG as well as the CME (Table 1) [58]. Consistent with this, the *CG33275 TRH265:GFP* enhancer expressed in fusion cells contains two TCGTG Dys/Tgo sites conserved in 12 *Drosophila* species. In summary, *CG33275* contains separable, but adjacent midline and tracheal enhancers, and the tracheal enhancer contains a subregion that drove expression restricted to fusion cells.

**Table 1:** DNA recognition sequence of PAS heterodimers.

Heterodimer	Name^1^	Sequence^2^
Sim/Tgo	CME	A*CGTG*
Trh/Tgo	CME	A*CGTG*
Dys/Tgo		T*CGTG*
Dys/Tgo		G*CGTG* 3
Dys/Tgo	CME	A*CGTG* 3
Sima/Tgo	HRE	R*CGTG*
Ss/Tgo	XRE	TNG*CGTG*
Per/Tim	E box	CA*CGTG*
Clock/Bmal	E box	CA*CGTG*

^1^ The names of the recognition sites are indicated: CNS midline enhancer (CME), hypoxia response element (HRE), xenobiotic response element (XRE) and the E box is the recognition site for bHLH proteins. Similar (Sima) is the fly hypoxia inducible factor-α, Spineless (Ss) functions in bristle, leg and antennal development and Period (Per), Timeless (Tim), Clock and Bmal function in circadian rhythms.

^2^ The CGTG core sequences shared by each recognition site are italicized.

^3^ The GCGTG and ACGTG sites are likely low affinity sites for Dys/Tgo [58].

### esg

**Figure 3:**
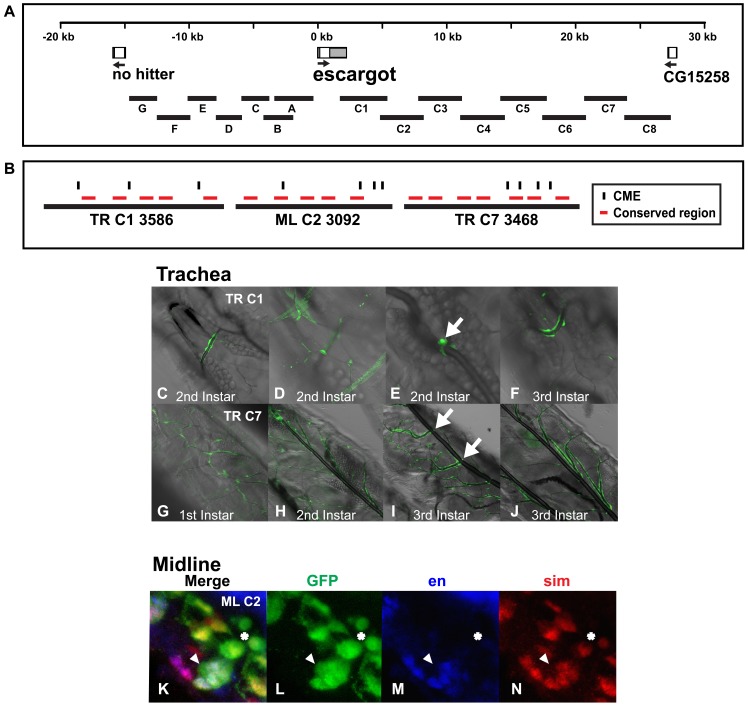
An *esg* genomic region contains a midline enhancer that is separable and distinct from two *esg* tracheal enhancers. (A) Genomic regions surrounding *esg* used to generate the reporter constructs and (B) the *esg* enhancers are shown as above. Gray boxes represent 5′ and 3′ untranslated regions. (C–J) Live larvae were analyzed by confocal and differential contrast microscopy and ventral views of the (C–F) *esg TR C1:GFP* and (G–J) *esg TR C7:GFP* reporters are shown. In larvae, the *esg TR C1:GFP* reporter is expressed sporadically in fusion cells (arrow in E) and the *esg TR C7:GFP* reporter is expressed in all tracheal branches and sporadically in the dorsal trunks (G–J), but consistently in fusion cells (arrows I). (K–N) Whole mount *esg ML C2:GFP* reporter embryos were stained with an anti-*GFP* antibody (green: L), *engrailed* monoclonal antibody (blue; M) and anti-*sim* antibody (red; N) and analyzed by confocal microscopy. The overlap in expression is shown in the merge image (K). Anterior midline glia express *GFP* and *sim* and are located dorsally within the nerve chord. Posterior midline glia that normally undergo cell death during this time can still be visualized with *GFP* (three cells surrounding star in N), but not *sim* or *engrailed*. The MNB and its progeny express *sim, engrailed* and *GFP* and are located ventrally within the nerve chord (arrowheads in K–N). Lateral view of a stage 16 transgenic embryo is shown; anterior is in the top, left hand corner and ventral is on the left.

*esg* is a zinc finger transcriptional repressor that regulates cell fate and development within the trachea and a subset of CNS cells, including the midline [35], [71]. *esg* is expressed at high levels in the embryo and moderate levels in the larval central nervous system, larval/adult midgut and adult testis [70], [72]. *esg* is rather isolated from other genes within the *Drosophila* genome and its next nearest upstream and downstream neighbors are ∼15–25 kb away (Fig. 3A). We examined this entire region to search for midline and tracheal enhancers and identified two, separable tracheal enhancers, *esg TR C1:GFP* (Fig. 3A–F) and *esg TR C7:GFP* (Fig. 3A, B and G–J) downstream of the coding sequence and another separable and distinct midline enhancer, *esg ML C2:GFP* (Fig. 3A, B and K–N), adjacent to and downstream of the *esg TR C1* tracheal enhancer. The endogenous *esg* gene is expressed in tracheal fusion cells during embryogenesis and first detected during branch migration [73]. The tracheal enhancers identified here drive expression only late in embryogenesis and during larval stages. The *esg TR C1:GFP* reporter is expressed sporadically in fusion cells (Fig. 3C–F), while the *esg TR C7:GFP* reporter is expressed in all tracheal branches and fusion cells and sporadically in the dorsal trunks (Fig. 3G–J).

In addition, the *esg ML C2:GFP* reporter drove a unique expression pattern in the midline, where it is expressed in both anterior and posterior midline glia and the median neuroblast and its progeny (Fig. 3K–N). This pattern is consistent with that of the endogenous *esg* gene, known to be expressed in a subset of mesectodermal and midline primordial cells [11]. In addition to these three enhancers, we found additional *esg* enhancers that drove expression in other embryonic tissues (Table S3).

### liprin *γ*

**Figure 4:**
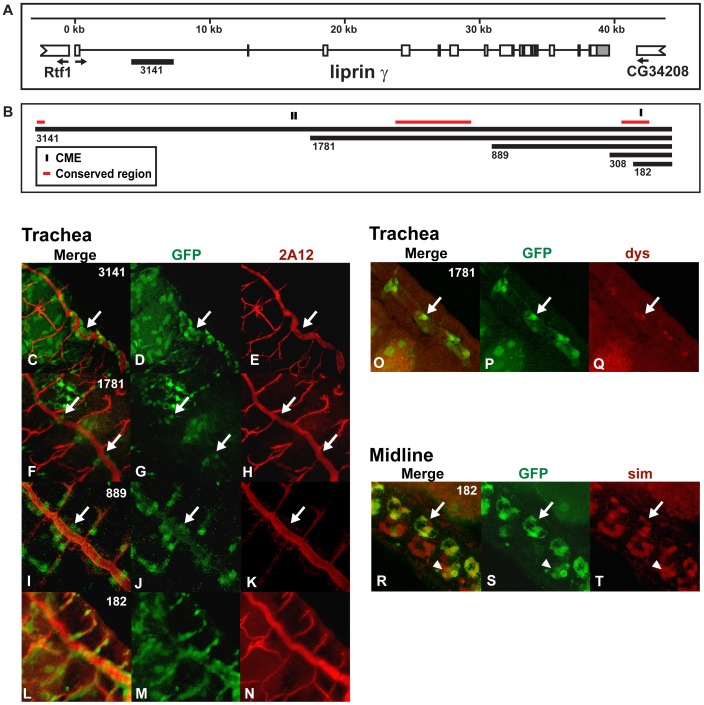
*Liprin γ* contains a conserved enhancer sufficient to drive expression in both midline glia and trachea. (A) The genomic regions within the first intron of *liprin γ* used to generate the reporter constructs and (B) the *liprin γ* enhancers are shown as in Fig. 1 and previously reported [59]. (C–T) Whole mount embryos were double-stained with an anti-*GFP* antibody (green: D, G, J, M, P and S) and monoclonal antibody *2A12* (red; E, H, K and N), anti-*dys* (red; Q) or anti-*sim* (red; T) and analyzed by confocal microscopy. The overlap in expression is shown in yellow in the merge column (C, F, I, L, O and R). Even though the *liprin γ 3141:GFP* and *liprin γ 889:GFP* reporters are expressed in the dorsal trunk (arrows C–E and I–K) and dorsal and ventral branches, *liprin γ 1781:GFP* is restricted to mostly fusion cells of the dorsal trunk (arrows F–H and O–Q). The *liprin γ 182:GFP* reporter is expressed in all tracheal cells (L–N), midline glia (arrows R–T) and a few midline neurons in certain segments (arrowheads R–T). Lateral views of stage 16 transgenic embryos are shown; anterior is in the top, left hand corner and ventral is on the left.

Liprin proteins interact with tyrosine phosphatases to regulate synapse formation. *Drosophila* contains three *liprin* genes and *liprin γ* is thought to antagonize the activity of the other two *liprins*: *α and β* at the synapse [74]. Our previous studies identified sequences within the *liprin γ* gene that drove expression in midline glia [59] and this same region drove expression in the embryonic and larval trachea (Fig. 4). This gene is expressed in both lateral and midline CNS glia at embryonic stage 14 [74] and several of the *liprin γ* reporter genes drove high levels of *GFP* expression during this stage and the remainder of embryogenesis. Both the *liprin γ 3141:GFP* (Fig. 4C–E) and *liprin γ 1781:GFP* (Fig. 4F–H) reporters drive expression in the dorsal trunk of the trachea, particularly within the posterior region of the embryo. The *liprin γ 889:GFP* reporter is expressed in additional tracheal cells, including the dorsal, visceral and lateral branches (Fig. 4I–K). Only the *liprin γ 1781:GFP* reporter drove expression in tracheal fusion cells (Fig. 4O–Q). The *liprin γ 308:GFP* reporter is expressed in the gut, but not in tracheal cells (data not shown), whereas the *liprin γ 182:GFP* reporter is expressed at high levels in all the trachea (Fig. 4L–N). In addition, this *liprin γ 182:GFP* reporter is sufficient to drive expression in midline glia and a few midline neurons, in a pattern that varies between segments (Fig. 4R–T). Therefore, the 182 bp core region contains a CME and conserved subregion that activates high levels of expression in both midline and trachea cells. Analysis of the expression pattern of the endogenous liprin *γ* gene indicates that it is either not expressed, or expressed at low levels, within the trachea during embryogenesis [70], [74]. This, taken together with 1) the high level of *GFP* expression in tracheal cells observed with the *liprin γ 182:GFP* reporter gene and 2) the diverse tracheal expression pattern of the larger *liprin γ* reporter genes, suggest that this region may only drive tracheal expression when isolated from surrounding sequences. Because multiple copies of the CME within a reporter gene, can drive expression in both the midline and trachea (see below), one function of sequences flanking the CME within enhancers is to limit expression of the gene to certain cell types. In summary, these experiments further define the minimal sequences needed for expression in the CNS midline within the previously identified *liprin γ* enhancer [59]. Moreover, these minimal sequences, when isolated from the genome and placed within reporter genes, can activate expression in tracheal cells as well.

### NetA and B

**Figure 5:**
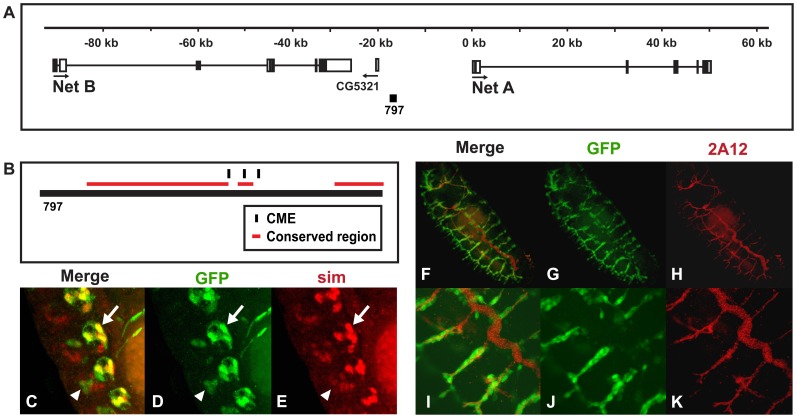
*Net* contains an enhancer that drives expression in both midline glia and trachea. (A) A genomic region located between *NetA* and *NetB* was used to generate a reporter construct and (B) the *Net* enhancer is shown. (C–K) Whole mount embryos were double-stained with an anti-*GFP* antibody (green: D, G and J) and anti-*sim* (red; E) or monoclonal antibody *2A12* (red; H and K) and analyzed by confocal microscopy. The overlap in expression is shown in yellow in the merge column (C, F and I). The *Net797:GFP* reporter drove expression in midline glia (arrows C–E; ganglionic branches of trachea are also visible in the image in green) and occasionally midline neurons within some segments (arrowheads C–E). The tracheal expression pattern of this reporter is unique in that *GFP* is high in tracheal cells, except cells within the dorsal trunk (F–K). Lateral views of stage 16 transgenic embryos are shown; anterior is in the top, left hand corner and ventral is on the left.

*NetA* and *NetB* are signaling molecules secreted by midline glia that attract axons to cross the midline and also function in glial migration [14], [15], [75], [76]. Both genes are expressed in many tissues, including midline glia [15], the larval trachea and adult nervous system [77]. The *Net797:GFP* reporter identifies a midline and tracheal enhancer located between *NetA* and *NetB* (Fig. 5A) that drove expression in midline glia (Fig. 5C–E) and trachea cells outside the dorsal trunk (Fig. 5F–K). This enhancer contains three CMEs and three highly conserved regions (Fig. 5B). Therefore, in contrast to the *CG33275* and *esg* enhancers and similar to the *liprin γ* enhancer described above, the single *Net* enhancer drove expression in both the midline and trachea. Moreover, the tracheal expression pattern provided by this enhancer is unique and highest in the visceral and dorsal branches and low or absent in the dorsal trunks (Fig. 5F–K). We also identified several *Net* enhancers that drive expression in tissues outside the midline and trachea (Table S3).

### comm

**Figure 6:**
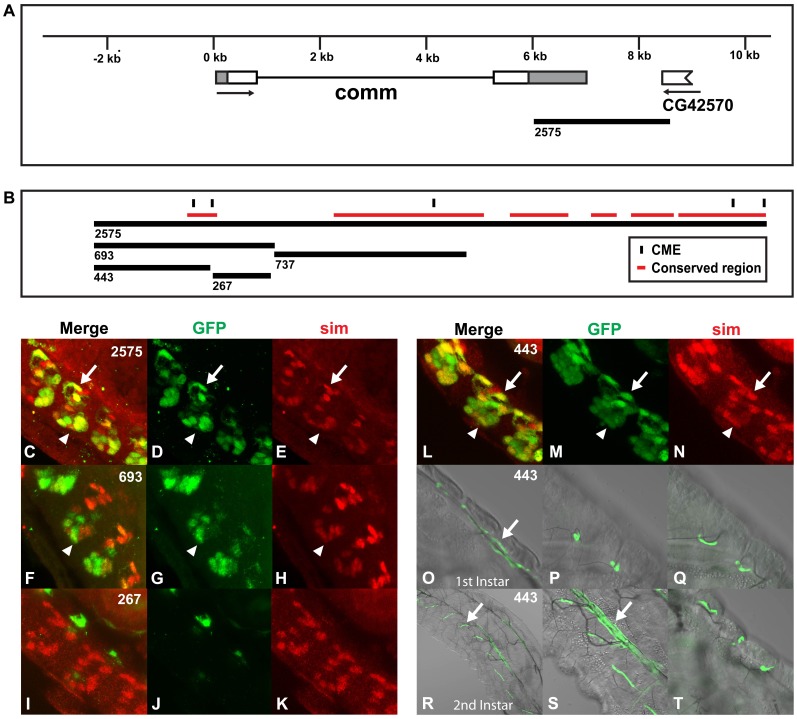
The *comm cis*-regulatory region contains an enhancer that drives expression in the embryonic midline and larval trachea. (A) Genomic regions surrounding and within *comm* were used to generate the reporter constructs and (B) the *comm* enhancer is shown. The closest gene upstream of *comm* is *CG6244*, which is 79,892 bp away. (C–N) Whole mount embryos were double-stained with anti-*GFP* (green: D, G, J and M) and anti-*sim* antibodies (red; E, H, K and N) and analyzed by confocal microscopy and the overlap in expression is shown in yellow in the merge column (C, F, I and L). (C–E) *comm2575:GFP* and (L–N) *comm443:GFP* are expressed in both midline glia (arrows) and midline neurons (arrowheads), while (F–H) *comm693:GFP* is restricted to some midline neurons (arrowheads). The *comm267:GFP* (I–K) and *comm737:GFP* (data not shown) reporters are not expressed in the midline. Lateral views of stage 16 transgenic embryos are shown; anterior is in the top, left hand corner and ventral is on the left. (O–T) *comm443:GFP* is also expressed in the tracheal dorsal trunks (arrows in O and S) as well as other tracheal branches (arrow in R). Live larvae containing the *comm443:GFP* reporter were analyzed by confocal and differential contrast microscopy and dorsal views are shown.

*comm* functions in synapse assembly and axon guidance by controlling the subcellular localization of membrane receptors. In particular, *comm* controls the *slit* receptor, *roundabout*, as CNS axons navigate the midline to ensure they cross the midline only once [20], [22], [78]–[80]. *comm* is expressed at high levels in midline glia and transiently in lateral CNS axons [80]. A midline enhancer is located in the 3′ untranslated region of *comm* (Fig. 6A and B), identified by testing *comm2575:GFP* (Fig. 6C–E), *comm737:GFP* (not shown), *comm693:GFP* (Fig. 6F–H), *comm443:GFP* (Fig. 6L–N) and *comm267:GFP* (Fig. 6I–K). All but the *comm737:GFP* and *comm267:GFP* reporters drove expression in midline cells. Each of the reporters that are active in the midline drive *GFP* expression in slightly different subsets of midline cells: *comm2575:GFP* is expressed in midline glia, with variable expression in midline neurons (Fig. 6C–E), *comm693:GFP* is expressed predominantly in a subset of midline neurons (Fig. 6F–H) and *comm443:GFP* is expressed in all midline cells (Fig. 6L–N). Only the *comm443:GFP* reporter drove expression in the trachea and tracheal expression initiated during early larval development and persisted throughout all larval stages (Fig. 6O–T). Therefore, *comm* contains a single enhancer that drives expression in both the embryonic midline and larval tracheal cells.

### moody

**Figure 7:**
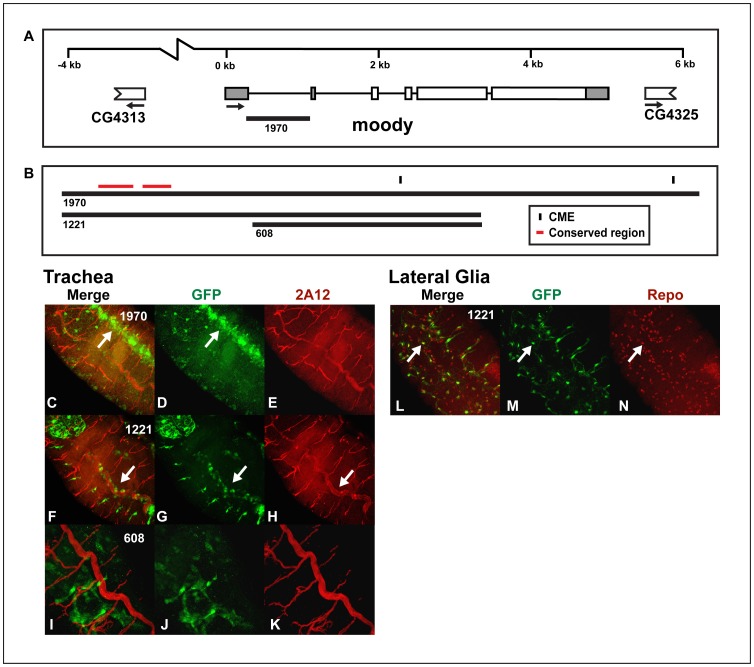
The *moody cis*-regulatory region contains a tracheal enhancer that overlaps with a lateral CNS glial enhancer. (A) The genomic regions surrounding and within *moody* used to generate the reporter constructs and (B) the *moody* enhancer is shown. (C–N) Whole mount embryos were double-stained with an anti-*GFP* antibody (green: D, G, J, and M) and monoclonal antibody *2A12* (red; E, H and K) or anti-*repo* monoclonal antibody (red; N) and analyzed by confocal microscopy. The overlap in expression is shown in yellow in the merge columns (C, F, I and L). The expression patterns of the (C–E) *moody1970:GFP*, (F–H and L–N) *moody1221:GFP* and (I–K) *moody608:GFP* reporters are shown. Both the *moody1970:GFP* (not shown) and *moody1221:GFP* (L–N) reporters also drive expression in lateral glia as indicated by co-localization with *repo* (arrows). Additionally, *moody 1221:GFP* is expressed in the dorsal trunk (arrows F–H), while *moody1970:GFP* is expressed in the dorsal vessel (arrows C and D) and lightly in the dorsal trunk. (C–H) Dorsolateral, (I–K) lateral or (L–N) ventral views of stage 16 transgenic embryos are shown; anterior is in the top, left hand corner.

*moody* is a rhodopsin and melatonin-like G-protein coupled receptor, found at the blood-brain barrier in adult flies [81] and that functions in germ cell migration in the embryo [82]. Moody is expressed in larval trachea, the larval/adult CNS, as well as many other tissues [77]. We tested three reporter genes: *moody1970:GFP, moody1221:GFP* and *moody608:GFP* (Fig. 7A and B) and found that *moody1970:GFP* is expressed in the dorsal vessel (Fig. 7C–E), but only *moody1221:GFP* drove expression in the dorsal trunks of the trachea, with expression highest in the posterior region of the embryo (Fig. 7F–H), similar to the *liprin γ*

*3141* enhancer (Fig. 4C–E). Also similar to the *liprin 3141:GFP* enhancer [59], the *moody1221:GFP* (Fig. 7L–N) and *moody1970:GFP* (not shown) enhancers are expressed in lateral CNS glia. *moody608:GFP* drove expression in the fat body (not shown), but is not expressed in the trachea (Fig. 7I–K). The identified *moody1221:GFP* tracheal enhancer contains two CMEs, although they are not highly conserved. This enhancer does not drive midline expression, rather sequences within the *moody* enhancer restrict expression to the trachea.

### Ect3

**Figure 8:**
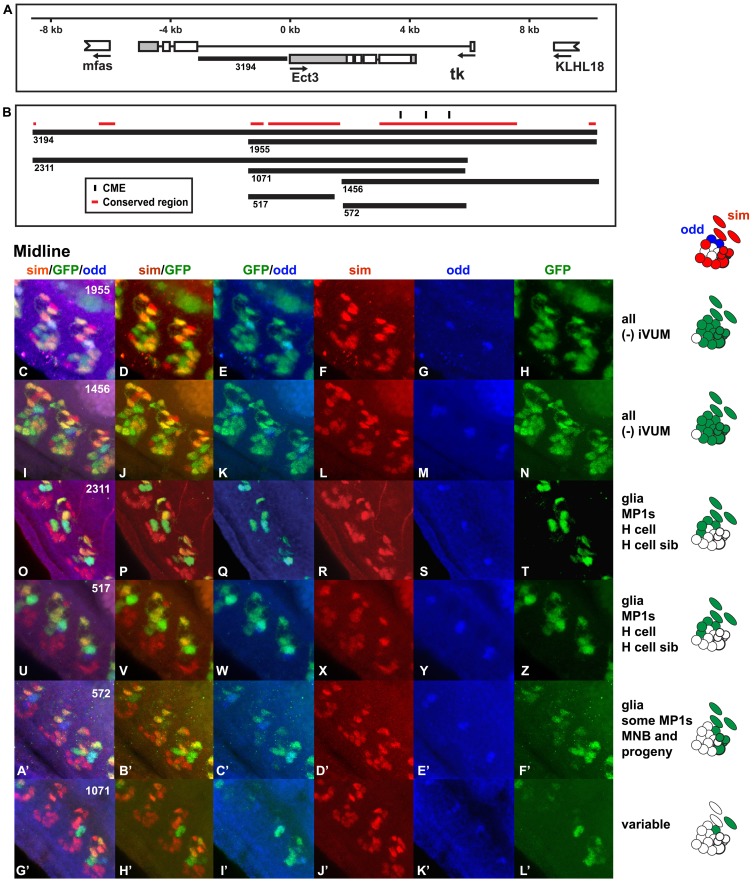
The *Ect3 cis*-regulatory region contains a midline enhancer sensitive to context. (A) The genomic region upstream of *Ect3* (and within the first intron of *Tk*) used to generate the reporter constructs and (B) the *Ect3* enhancers are shown. (C–L’) Whole mount embryos were stained with anti-*sim* (red; F, L, R, X, D’ and J’), anti-*odd* (blue; G, M, S, Y, E’ and K’) and anti-*GFP* (green: H, N, T, Z, F’ and L’) antibodies and analyzed by confocal microscopy. The overlap in expression is shown in the merge columns: all three antibodies (C, I, O, U, A’ and G’), *sim* and *GFP* (D, J, P, V, B’ and H’) and *GFP* and *odd* (E, K, Q, W, C’ and I’). Odd is expressed only in MP1 midline neurons. Both *Ect3 1955:GFP* (C–H) and *Ect3 1456:GFP* (I–N) drive expression in the all midline cells, with the exception of a single iVUM. *Ect3 2311:GFP* (O–T) and *Ect3 517:GFP* (U–Z) are restricted to some midline glia, MP1 neurons, the H cell and H cell sib. *Ect3 572:GFP* (A’–F’) is expressed in midline glia, some MP1 neurons and some of the MNB and its progeny. Finally, *Ect3 1071:GFP* (G’–L’) drives only spotty midline expression. Lateral views of stage 16 transgenic embryos are shown; anterior is in the top, left hand corner and ventral is on the left. The midline expression pattern of each reporter is shown schematically on the right.

The Ect3 protein is a galactosidase expressed in midline glia, [11] as well as other tissues, that regulates autophagic cell death [83]. Because *Ect3* is located within the first intron of *Tachykinin (Tk)*, the identified midline enhancer is found just upstream of *Ect3* as well as within the first intron of *Tk* (Fig. 8A and B). *Tachykinin* (*Tk*) is a neuropeptide hormone expressed at high levels during 18–24 hours of embryogenesis, early larval stages and in the adult male [77]. The midline enhancer identified here likely regulates expression of the endogenous *Ect3* gene, because only *Ect3,* and not *Tk,* is expressed in the embryonic midline [11], [70]. This midline enhancer is sensitive to small changes in sequence, such that various subregions drive different midline expression patterns. The *Ect3 3194:GFP* reporter contains the region bordered by the first exon of *Tk* on the 5′ end and the *Ect3* transcription start site on the 3′ end (Fig. 8A and B). This reporter (not shown), as well as the *Ect3 1955:GFP* (Fig. 8C–H) and *Ect3 1456:GFP* (Fig. 8I–N) reporters drive high levels of *GFP* expression in all midline cells, with the exception of one iVUM neuron. Four other reporters are expressed in a more limited set of midline cells: 1) the *Ect3 2311:GFP* (Fig. 8O–T) and 2) *Ect3 517:GFP* (Fig. 8U–Z) reporters drove expression in midline glia, MP1 neurons, the H-cell and spotty and variable expression in the H-cell sib, while the 3) *Ect3 572:GFP* (Fig. 8A’-F’) reporter drove expression in some midline glia, MP1 neurons and some of the progeny of the MNB and 4) the *Ect3 1071:GFP* (Fig. 8G’-L’) reporter drove spotty and variable expression in only a few midline cells within some segments. Taken together, the data indicate the *Ect3* enhancer contains sequences that promote expression in all midline cells. The endogenous *Ect3* gene is expressed in midline glia [11] and all of the *Ect3* reporters drive expression in these cells. The 572 bp *Ect3* enhancer contains three CMEs and a highly conserved subregion that can combine with another, downstream subregion located within both the *Ect3 1955:GFP* and *Ect3 1456:GFP* reporters, to enhance expression in certain midline cells. In addition, the 517 bp region can drive expression in midline cells, despite the absence of any CMEs. Therefore, this region of the genome contains multiple subsections that combine to drive expression in midline cells.

In summary, ten enhancers were identified: six of the enhancers drove expression in the midline, seven in the trachea and three in both the midline and trachea (Table 2). *CG33275* and *esg* each contain adjacent, separable midline and tracheal enhancers; whereas *liprin γ, Net* and *comm* each contain one enhancer that drove expression in both the midline and trachea. The *moody* enhancer drove expression only in the trachea and the *Ect3* enhancer drove expression only in the midline. Despite providing expression in overlapping cell types, each enhancer drove a unique expression pattern within the midline and trachea. Next, these enhancers, together with previously reported enhancers discovered by several groups, were combined to search for overrepresented motifs that may correspond to binding sites for transcription factors that activate or repress genes in the midline and/or trachea.

**Table 2:** Ten identified midline and tracheal enhancers.

Gene	Size^1^	Position^2^	Tissue	Midline cells^3^	Tracheal cells^4^
*CG33275*	753	exon 5+ intron 5	midline	glia (st. 13)	**–**
	1297/265	intron 5	trachea	**–**	all (st. 11)/fusion cells (st. 13)
*esg*	3092	∼ 5 kb downstream	midline	glia, MNB and progeny (st.12)	**–**
	3586	downstream	trachea	**–**	larval fusion cells (1^st^ instar)
	3468	∼ 20 kb downstream	trachea	**–**	larval fusion cells; secondary branches (1^st^ instar)
*liprin γ*	889/182	intron 1	both	glia (st. 12)/glia and sporadic in MNB and progeny (st. 12)	DT(st. 15)/all (st. 13)
*Net*	797	∼ 10 kb downstream	both	mostly glia (st. 12)	all but DT (st. 12)
*comm*	443	3′ untranslatedregion	both	all midline cells (st. 10)	larval dorsal trunk and some secondary branches (st. 17)
*moody*	1221	intron 1	trachea	**–**	posterior DT (st. 15)
*Ect3*	517	upstream of *Ect3*/intron 1 of *Tk*	midline	glia, MP1s, H cell and H cell sib (st. 10)	**–**

^1^ The size of the minimal fragment with enhancer activity,

^2^ the position of the enhancer relative to the gene and^ 3^midline and ^4^tracheal cells that exhibit enhancer activity are indicated.

^3,4^ The stage of development when reporter expression is first observed is indicated in parentheses. The absence of expression in the midline or trachea is indicated with a dash.

### Proximal CME Sequences

A longterm goal is to use the midline and trachea as models to study how transcription factors combine with cell type specific co-factors to regulate unique gene sets, and, in this way, dictate development of unique tissues. Including the ten enhancers identified here, nineteen different midline enhancers and nineteen tracheal enhancers have been identified. To identify sequences that promote or inhibit CME utilization in either the midline or trachea, we analyzed the enhancers in two different ways. First, we searched sequences directly flanking the CME within defined enhancers to determine if these sequences could predict whether a particular CME is utilized by Sim/Tgo or Trh/Tgo and secondly, we searched the smallest region sufficient to drive expression in a tissue for reiterated motifs that may help restrict or promote gene expression in the midline and trachea.

Results from this analysis indicate that the nucleotide located both immediately upstream and downstream of the CME are strong, but not absolute, determinants of whether the CME is utilized in the midline or trachea (Table 3). We found sixty-six CMEs within all the enhancers examined here and 34/66 consisted of the sequences AACGTGC, TACGTGA or TACGTGC (CME underlined), while the sequences AACGTGG, GACGTGT, TACGTGG were not found in any of the enhancers, suggesting that Sim/Tgo and Trh/Tgo may not bind these sequences (Table S4). Enhancers that drive only midline expression, most often contain the sequence (A/G/T)ACGTGC, while enhancers that solely drive tracheal expression contain the sequence (A/T)ACGTG(A/C/T) and enhancers that function in both the midline and trachea, most often contain the consensus (A/T)ACGTGC (Table 3). Therefore, the nucleotides immediately flanking the core CME may be one determinant that controls if a PAS heterodimer will bind this sequence within different cell types. We further investigated this by constructing and testing the expression pattern of synthetic reporter genes.

**Table 3:** Proximal CME context in midline and tracheal enhancers.

Mostcommon^1^	Number^2^	Neverfound^3^	Tissue^4^	PredictedConsensus^5^
A ACGTG C	14	A ACGTG G	midline	(A/G/T) ACGTG C
T ACGTG A	10	G ACGTG T	trachea	(A/T) ACGTG (A/C/T)
T ACGTG C	10	T ACGTG G	both	(A/T) ACGTG C

Sixty-six CMEs were found in all midline and tracheal enhancers examined. ^1^The nucleotides found directly 5′ and 3′ of the CME within the enhancers and ^2 ^the number of times that sequence was found in all the midline and tracheal enhancers are listed. The three sequence contexts found in the left column represent 52% of the CMEs found within all enhancers (34/66; Table S4), while ^3^other sequence contexts were not found in any enhancers. ^4, 5^Sequences flanking the sixty-six CMEs were used to derive a consensus sequence for genes expressed in the midline, trachea or both tissues.

### Synthetic Genes

Enhancers are modular and contain multiple binding sites for many activators and repressors that work together in large multi-protein complexes to regulate transcription in different cell types. Nevertheless, individual binding sites of a limited number of transcription factors are sufficient to drive expression in certain tissues, particularly when present in more than one copy. Relevant to this study, four copies of the CME fused to β*-galactosidase* or *GFP*, is sufficient to drive reporter expression in both the midline and trachea [7], [8], [59], [63]. Our previous results indicated that the context surrounding the CME within such multimerized constructs had a large impact on the reporter gene expression pattern [59]. To confirm and extend the results obtained with endogenous enhancers, we analyzed the expression pattern of additional synthetic reporters (Table 4). The synthetic sequences were modifications of CMEs derived from either the *wrapper* (*synth 1–4 and synth 6*) or *Toll* midline enhancers (*synth 5, Toll and synth 7–12*). We chose these particular sequences because both had been tested previously within synthetic reporter genes and drove different patterns of expression. The CME and flanking sequences found in the *wrapper* enhancer, when multimerized four times, drives expression only in the midline [59], while the CME and flanking sequences found in the *Toll* enhancer drives expression in both the midline and trachea [62].

**Table 4:** Sequence of synthetic reporter constructs.

**Midline only**	
synth 1	ATTACACTCTCCGCTTCAGAGAACGTGCTGCTGTTGCATATTCCGA
synth 2	TTCAGAGAACGTGCTGCTGTTGCATATTCCGAGATAAAATGTCATTGT
synth 3	GCGACACTCTCCGCTTCAGAGAACGTGCTGCTGTTGCATATTCCGA
synth 4	ATTACACTCTCCGCTTCAGAGAACGTGCTGCTTAAAA
synth 5	TATGCACAATGACATTTAGCAGAAATTCAGACGTGCCACAGACCA
**Neither Midline nor Trachea**	
Sox	CACAATGACGTGCCACAGA
synth 6	ATTACACTCTCCGCTTCAGAGAACGTGCTGCTGGCGCATATTCCGA
**Both Midline and Trachea**	
Toll	AAATTTGTACGTGCCACAGA
synth 7	AAATTTGTACGTGCCACAGA**TAATTA**
synth 8	AAATTTGTACGTGCCACAGA**GTTGCAT**
synth 9	AAATTTGTACGTGCCACAGAGG**CGTGGGAAC**CGAGCTGAAAGTAA**GTTTCTCACA**CA
synth 10	ACAATGACATTTCAGACGTGCCACA
**Trachea only**	
synth 11	AAATTTGTACGTGCTTTTTATCTCTGAAGCGGAGAGTGTAAT
synth 12	AAATTTGTACGTGCCACAGAGGATGCACCCACGAGCTGAAAGTAATGGGCCACCA

**Figure 9:**
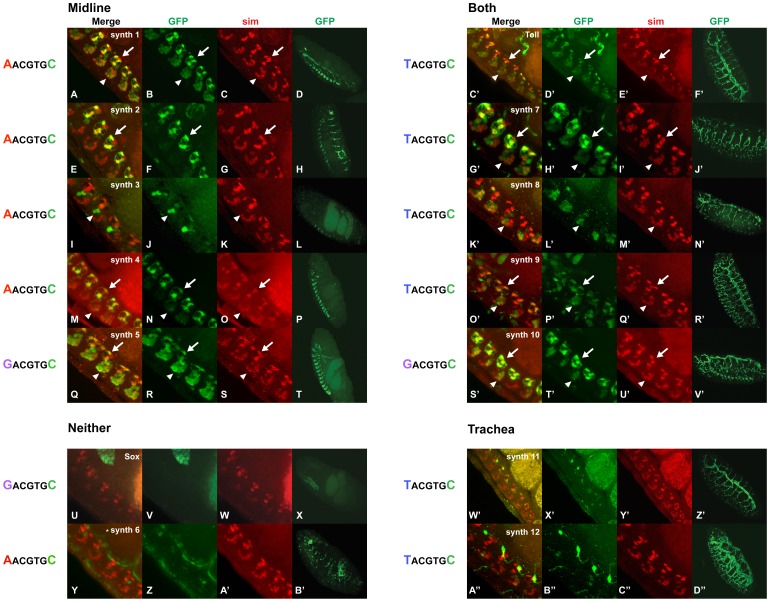
Proximal sequence context flanking the CME contributes to the midline and tracheal expression pattern. Whole mount transgenic embryos containing one of the multimerized synthetic reporter constructs were labeled with anti-*GFP* (green; B, D, F, H, J, L, N, P, R, T, V, X, Z, B’, D’, F’, H’, J’, L’, N’, P’, R’, T’, V’, X’, Z’, B’’ and D’’) and anti-*sim* (red; C, G, K, O, S, W, A’, E’, I’, M’, Q’, U’, Y’ and C’’) antibodies and analyzed by confocal microscopy. The overlap in expression between *GFP* and *sim* is yellow (merge; A, E, I, M, Q, U, Y, C’, G’, K’, O’, S’, W’ and A’’). Midline *GFP* expression is driven by the (A–D) *synth 1:GFP*, (E–H) *synth 2:GFP* (I–L) *synth 3:GFP*, (M–P) *synth 4:GFP* and (Q–T) *synth 5:GFP,* whereas the (U–X) *Sox:GFP* and (Y–B’) *synth 6:GFP* reporters are not expressed in either the midline or trachea. The (C’-F’) *Toll:GFP,* (G’-J’) *synth 7:GFP*, (K’-N’) *synth 8:GFP*, (O’-R’) *synth 9:GFP* and (S’-V’) *synth 10:GFP* synthetic reporters are expressed in both the midline and trachea, whereas the expression pattern of the (W’-Z’) *synth 11:GFP* and (A’’-D’’) *synth 12:GFP* reporters are restricted to trachea only. The expression patterns of the *Toll:GFP* and *Sox:GFP* reporters were previously reported [59]. Note that midline *GFP* expression driven by the (A–D) *synth 1:GFP*, (M–P) *synth 4:GFP*, (Q–T) *synth 5:GFP,* (C’-F’) *Toll:GFP,* (G’-J’) *synth 7:GFP*, (O’-R’) *synth 9:GFP* and (S’-V’) *synth 10:GFP* reporters is in both neurons and glia, whereas expression driven by the (E–H) *synth 2:GFP* reporter is restricted to midline glia and expression of the (I–L) *synth 3:GFP* and (K’-N’) *synth 8:GFP* reporters is restricted to midline neurons**.** The immediate CME context within each synthetic sequence is indicated to the left of the images and the entire sequence of each synthetic reporter construct is listed in Table 4. Arrows indicate midline glia and arrowheads indicate midline neurons. Lateral or ventrolateral views of stage 16 transgenic embryos are shown; anterior is in the top, left hand corner and ventral is bottom, left. Four copies of each synthetic sequence were tested within the reporter constructs.

Sequences of synthetic constructs multimerized four times and fused to *GFP* within reporter constructs are listed according to the tissue that expressed each synthetic reporter (Fig. 9). The CME is enlarged within each sequence. Sites important for midline expression within the *wrapper* enhancer [67] are underlined in *synths 1* **–** *6* and include putative binding sites for Sox (ATTGT), *pointed* (CTCTCCG) and unknown (AAAA) transcription factors. Binding sites for engrailed (TAATTA), Vvl (TTGCAT) and Suppressor of Hairless (**GTGGGAAC** CGAGCTGAAAGTAAG**TTTCTCAC** **)** were added to the *Toll* CME sequence and shown in bold in *synths 7* **–** *9*.

To understand which sequences within the previously published synthetic constructs are responsible for the two different expression patterns, we tested additional synthetic reporter genes. The sequence context surrounding the CME in the *wrapper* enhancer was tested using two approaches. First, the 70 bp minimal *wrapper* enhancer was divided into two sections and tested independently: *synth 1* contained sequences 7–53 and *synth 2* contained sequences 28–70 of the *wrapper* minimal enhancer. Both of these constructs contain the single CME and flanking sequences found in the *wrapper* enhancer and both of these multimerized reporters were expressed in the midline, but not the trachea (Fig. 9A–H). However, the expression pattern within the midline differed and *synth 1* drove expression in all midline cells (Fig. 9A–C), while *synth 2* drove expression restricted to the midline glia (Fig. 9E–G). Next, we tested specific sequences within these constructs. When the ATTA sequence found at the 5′ end of *synth 1* is changed to GCGA within *synth 3*, the reported gene is still expressed in the midline, but only in 1–3 cells per segment (Fig. 9I–L), suggesting this may have created a repressor binding site that limits midline expression. In contrast, changing the 14 nucleotides found at the 3′ end of *synth 1* (GTTGCATATTCCGA) to TAAAA within *synth 4*, had only a small effect on the midline expression pattern of *GFP* (compare *synth 1* in Fig. 9A–C with *synth 4 *in Fig. 9M–O), while changing the first three nucleotides within this 14 bp region from GTT found in *synth 1* to GGC within *synth 6* almost completely eliminated midline expression (Fig. 9Y–B’).

As mentioned above, the multimerized *Toll* CME and flanking sequences drives reporter expression in all midline and tracheal cells [62] (Fig. 9C’–F’). We tested whether adding binding sites of known midline transcription factors affected the expression pattern of this synthetic reporter gene. For this, an Engrailed binding site (TAATTA; [84]) was added to *synth 7*, a binding site for the POU domain transcription factor, Vvl (GTTGCAT; [64]) was added to *synth 8* and binding sites for the Suppressor of Hairless transcription factor (C**GTGGGAAC**CGAGCTGAAAGTAAG**TTTCTCAC**ACA; [85]) within *sythn 9* (Table 4). Surprisingly, none of these changes in sequence affected the expression pattern of the *Toll* CME reporter and all of the reporters were expressed in the trachea (Fig. 9C’–R’), although *synth 7*, was expressed at a lower level in the dorsal trunks relative to the rest of the trachea; a pattern not observed with the other reporters (Fig. 9J’). These nucleotide changes also did not eliminate the midline expression pattern, although *synth 8,* containing the Vvl binding site, drove expression in midline neurons, but not midline glia (Fig. 9K’–M’).

In summary, four of five synthetic constructs containing the sequence AACGTGC, were expressed in midline cells only (Fig. 9A–P and Table 4), while the fifth was not expressed in either the midline or trachea (Fig. 9Y–B’). Four of six synthetic constructs containing the related sequence, TACGTGC, drove expression in both midline and tracheal cells (Fig. 9C’–R’) and the other two drove expression only in trachea (Fig. 9W’–D’’). Finally, three synthetic genes containing the sequence, GACGTGC, each exhibited a different expression pattern: *synth 5* was expressed only in the midline (Fig. 9Q–T); *Sox* in neither tissue (Fig. 9U–X) and *synth 10* in both the midline and trachea (Fig. 9S’–V’), suggesting that this sequence is more sensitive to effects of additional sequences flanking the CME, compared to the other contexts. Taken together, the results suggest that the nucleotides immediately upstream and downstream of the CME had the largest impact on whether *GFP* was expressed in the midline or trachea. In most cases, the spacing and sequences between the CMEs did not affect whether or not the synthetic reporter was expressed in the midline or trachea, but instead, these sequences controlled which cell types within the midline or trachea, expressed *GFP*. These results, together with those of the endogenous enhancers suggest that sequences proximal to the CME are strong, but not absolute, predictors of midline or trachea expression. Additional sequences, more distal to the CME, also impact CME utilization, as well as control which cell types express the gene.

### Identification of Overrepresented Midline and Tracheal Motifs

**Figure 10:**
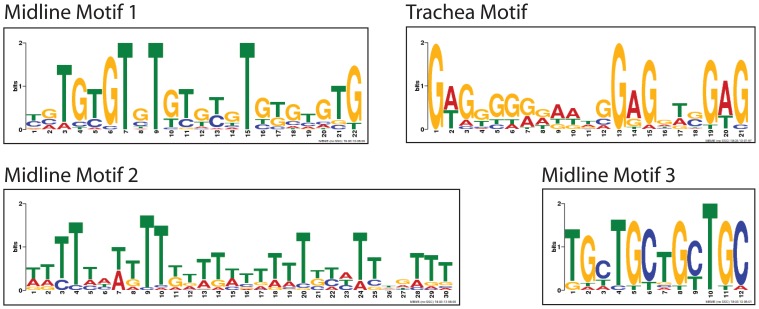
Motifs overrepresented in midline and tracheal enhancers identified with MEME. MEME [61] was used to identify motifs overrepresented in midline and tracheal enhancers. The expected number of motifs one would find in a similarly sized set of random sequences (E-value) and the number of times each site was found within the enhancers are indicated in Table 5. Each motif was identified using two, related data sets (see text).

To identify motifs other than the CME overrepresented within midline and tracheal enhancers, we used MEME (http://meme.ebi.edu.au/meme/cgi-bin/meme.cgi; [61]). Examination of enhancers that drive expression in both tissues together with enhancers that drive expression only in the midline identified three overrepresented midline motifs (Fig. 10 and Table 5). In addition, MEME analysis of enhancers expressed in both tissues together with enhancers that drive expression only in the trachea, led to the identification of a single overrepresented tracheal motif. All four motifs consist of simple sequence repeats: midline motif 1 is 22 bp long, consists of repeating TG residues and is present 18 times in the 19 midline enhancers; midline motif 2 is 31 bp, consists mostly of T residues and is found 50 times; and midline motif 3 is 12 bps, consists of four repeats of the trinucleotide TGC and is found 25 times. The identified tracheal motif is 22 bp long, consists mostly of G residues and is found 16 times in the 19 tracheal enhancers examined. To ensure these results were not biased by including enhancers of variable sizes (336–3586 bp), we compared the above results to those obtained after restricting the search to only the smallest midline and tracheal enhancers identified, and excluded enhancers that function in both tissues. The same motifs were identified using this approach.

**Table 5:** Midline and tracheal motifs identified with MEME.

Motif Name	^3^Number of enhancers examined	^4^Number of enhancers containing site	^5^E value	^6^Total number of sites	^7^Length of site
^1^midline 1	19	9	1.2 e-05	18	22
^1^midline 2	19	14	7.2 e-34	50	31
^1^midline 3	19	12	1.2 e-10	25	12
^2^trachea 1	19	8	1.4 e-10	16	22

MEME analysis was used to identify motifs overrepresented in midline and tracheal enhancers. Three motifs were found in midline enhancers and one in tracheal enhancers (Fig. 10). Results from^1^twelve enhancers that drive expression in the midline together with seven enhancers that drive expression in the midline and trachea or ^2^twelve enhancers that drive expression in the trachea and seven enhancers that drive expression in the midline and trachea are shown, as well as.^ 3^the number of enhancers examined,^ 4^number of enhancers containing the motif,^ 5^likelihood of finding the motif by chance,^ 6^number of times the site was found in all the enhancers examined and.^ 7^length of the identified site.

## Discussion

DNA sequences located within introns and intergenic regions are known to regulate transcription and package DNA; however, many aspects of these processes remain unknown. Enhancers that control gene expression patterns are modular and contain binding sites for transcription factors that function in a combinatorial manner [86]–[90]. The array of transcription factors expressed within a particular cell type, and available to bind enhancers, depends upon the cell’s position in the embryo as well as its developmental history. Identifying shared properties of enhancers active within a given cell type is challenging because most genes display their own unique expression pattern. Moreover, transcription factor binding sites can be combined in multiple ways to generate a similar expression pattern [91]. As a result, the complexity of gene expression patterns is often reflected by a complex and unpredictable organization of *cis*-regulatory sequences. Untangling this complexity to reveal how enhancers integrate positional, environmental and physiological information to regulate gene expression is needed to understand how organisms adapt to their internal and external environments at the molecular level.

Each enhancer described here contained a unique constellation of transcription factor binding sites and, as a result, drove a unique expression pattern in midline and tracheal cells. By analyzing and comparing available midline and tracheal enhancers, we have identified sequences, both proximal and distal to the CME, which promote expression in one tissue or the other. These reporters can be exploited in the future to identify transcription factors that bind to the enhancers using techniques such as chromatin immunoprecipitation, the yeast one hybrid assay and mutant genetic backgrounds. In addition, over one thousand *GAL4* lines have been identified that drive expression in embryonic midline cells [92], providing a rich resource for extending these studies.

We have identified enhancers that drive expression restricted to midline glia, midline neurons, all embryonic tracheal cells, tracheal fusion cells, the posterior dorsal trunk, lateral tracheal branches, terminal cells or larval trachea cells and are activated at different stages of development (Table 2). In addition to identifying new motifs that may bind characterized or novel transcription factors, these studies provide tools for expressing transgenes in specific midline and tracheal subtypes for experimental purposes. When combined with toxins, RNAi or fluorophores, these sequences can be used to ablate cells, knockdown expression of specific genes and/or specifically label midline or tracheal subtypes. Moreover, genes within orthologous vertebrate tissues, such as glia and blood vessels, are regulated by similar regulatory networks [6]. Comparing midline and tracheal regulatory networks with networks that impact related tissues in other organisms will reveal how functionally distinct tissues are generated.

### Midline and Tracheal Enhancer Motifs

Several families of transcription factors contain members that bind related, but slightly different DNA recognition sequences. Examples include members of the nuclear receptor family (reviewed in [93]) and bHLH proteins [94], [95]. Nuclear receptor homodimers and heterodimers bind DNA response elements consisting of two inverted repeats separated by a trinucleotide spacer. Specificity is determined by interactions between protein loops on the second zinc finger of a particular steroid receptor DNA binding domain and the trinucleotide spacer within the DNA recognition site [96], [97]. Similarly, the recognition sequence of bHLH transcription factors is called the E box and consists of the sequence CANNTG [98]. Specific bHLH heterodimers preferentially bind E boxes containing various internal dinucleotides (represented by the NN within the E box) [99]. The bHLH-PAS proteins investigated here are a subfamily within the bHLH superfamily of transcription factors. The PAS domain helps stabilize protein-protein interactions with other PAS proteins, as well as with additional co-factors, some of which mediate interactions with the environment [53]–[55]. The evolutionary relationship of bHLH and bHLH-PAS proteins is also reflected in the similarity of their DNA recognition sequences. The CME is related to the E box and historically has been considered to consist of a five rather than six base pair consensus (Table 1). Previous results indicated bHLH-PAS heterodimers strongly prefer the internal two nucleotides of the binding site to be “CG”, while the nucleotide immediately 5′ to this core helps to discriminate which heterodimer binds the site. The first crystal structure of a bHLH-PAS heterodimer bound to DNA reveals that the recognition sequence of the human Clock/Bmal bHLH-PAS heterodimer actually consists of seven base pairs, rather than five [100]. This is consistent with results reported here that suggest Sim/Tgo and Trh/Tgo heterodimers preferentially bind highly related, but slightly different seven base pair sequences (Tables 3 and 4). In addition, experiments with fly Sim and human Tgo, called Aryl hydrocarbon receptor nuclear translocator protein (Arnt), using the Systematic Evolution of Ligands by Exponential Enrichment (SELEX) approach, identify the sequence DDRCGTG (D = A, C or T and R = either purine) as the Sim/Tgo binding site [101]. Our results agree with this, although the consensus sequence we identify by examining known enhancers, is shifted by one nucleotide (DACGTG C; Table 3). In the midline and tracheal enhancers, we found sixty-six copies of the CME, ACGTG, and forty-eight copies of the related sequence, GCGTG, also identified in the SELEX experiments (Table S5). Half of these GCGTG sites fit the seven bp consensus TGCGTGR and future experiments are needed to determine their importance within the various enhancers. Our results indicated that the CME context favored within midline and tracheal enhancers as well as enhancers active in both tissues, was very similar (Table 3), yet clearly distinct from binding sites of other bHLH and bHLH-PAS heterodimers (Table 1). Based on the expression pattern of certain reporter genes examined here, the same CME may be bound by Sim/Tgo in the midline and Trh/Tgo in the trachea within certain enhancers. Within other contexts, the CME appears to be discriminated by these different heterodimers, because some enhancers drive expression in only one tissue or the other.

### Enhancer Complexity

Results from both endogenous enhancers and the synthetic reporter genes confirm the importance of the proximal sequences in limiting expression to either the midline or trachea. While the proximal context of the CME plays a role, additional sequences clearly combine with the CME to ultimately determine if an enhancer is functional in the midline or trachea. Taken together, these results indicate that proximal motifs combine with additional sequences not only to determine whether or not a gene is expressed in the midline or trachea, but also to determine which cellular subtypes express the gene and when it is activated within a tissue. Future experiments will reveal if 1) changing the sequence, AACGTGC, to TACGTGC within a midline enhancer will cause the enhancer to drive expression in trachea as well and 2) if changing the sequence, TACGTGC, to AACGTGC within an enhancer that drives expression in both the midline and trachea, will restrict expression to only the trachea. Sequences proximal to the CME likely affect the affinity of either Sim/Tgo and/or Trh/Tgo heterodimers to the DNA, but binding sites for additional factors that interact cooperatively to stabilize an entire transcription complex are needed for high levels of expression within a particular cell. Moreover, recent experiments indicate that enhancers containing multiple CMEs are activated earlier in the embryonic midline than enhancers containing only one CME [102]. The authors of this study suggest Sim/Tgo binding sites may be sufficient for activation in the early embryo, but that binding sites for additional transcription factors must combine with the CME to drive expression within the later, more complex embryo.

The experiments described here as well as previous experiments indicate that the CME is not always necessary for either midline or tracheal expression. A number of enhancers that drive expression in both tissues do not contain a CME, including: 1) a 517 bp autoregulatory *Vvl* enhancer that drove expression in both the midline and trachea [64], 2) another, separate tracheal enhancer of *Vvl* [34], 3) a *trh* autoregulatory enhancer 4) the *link* enhancer, after its sole CME has been destroyed [68], 5) a *dys* tracheal enhancer [58], 6) a tracheal enhancer of *CG15252*, 7) a tracheal enhancer of *CG13196*, and 8) the 517 bp *Ect3* midline enhancer described here (Fig. 8). These sequences may be capable of driving midline and tracheal expression due to the presence of unknown, low affinity binding sites for Sim/Tgo and Trh/Tgo, or binding sites for other midline and tracheal transcription factors that can help recruit PAS heterodimers to the enhancer. To understand how a combination of binding sites that does not include the CME can drive expression in the midline and trachea, as well as how CMEs are distinguished by Sim/Tgo and Trh/Tgo heterodimers, we searched and found other regulatory motifs, both proximal and distal to the CME in midline and tracheal enhancers. Future experiments are needed to understand how Sim and Trh interact with additional factors to modify chromatin structure, and ongoing mutagenesis experiments will help reveal roles for the identified T, TG and G rich regions within midline and tracheal enhancers (Fig. 10). These repetitive motifs are found scattered throughout the enhancers and do not appear to have a fixed location relative to the CMEs. AT rich regions bend and denature relatively easily, facilitating DNA looping and are often found in *cis*-regulatory regions. The short, repetitive regions identified here may interact with specific transcription factors, such as Sox, Forkhead-type or other remodeling proteins to open chromatin [103], [104]. Alternatively, these regions may be involved in 1) recruiting transcription factors after replication, 2) nucleosome positioning and/or 3) binding of histone modification enzymes to enhance transcription; all of which may affect quantitative and qualitative genetic variation in expression [105]. In addition, results with multiple transgenic lines indicate the synthetic constructs show little variation in patterns and levels and consistently recruit Sim/Tgo and/or Trh/Tgo regardless of insertion site. This suggests that factors interacting with these relatively small multimerized sequences (20–57 bp) are sufficient to open chromatin to allow for efficient transcription. Taken together, results from a number of labs suggest the following enhancer characteristics combine to determine if a gene will be expressed in the midline or trachea: 1) the number of CMEs within the enhancer, 2) the proximal context surrounding each CME and 3) binding sites for additional activators, repressors and/or factors that affect chromatin structure.

### Evolution of Sim and Trh Developmental Functions

While these experiments focus on the *cis*-regulatory sequences that control the expression of genes within the midline and trachea, they do not address why many genes are expressed in both of these tissues and regulated by related PAS heterodimers. It is predominantly genes expressed in the CNS midline glia, rather than the midline neurons, that are also expressed in tracheal cells. PAS proteins perform diverse functions across all biological kingdoms and most characterized members function as environmental sensors [53]–[55]. Historically, Sim and Trh have been considered exceptions and their developmental functions have been emphasized [106]. However, functions of Sim and Trh may have arisen in ancestral organisms that more closely resemble the adult form of *Drosophila*, a stage when Sim and Trh may function more similarly. For instance, in adult flies, both glia and trachea provide support and energy to neurons and *trh* is expressed in the CNS late in embryogenesis and throughout the remainder of the fly’s life. In the adult fly brain, tracheal development is guided by glial cells, and ablating glia causes the trachea to branch more extensively within this tissue [107]. Related mechanisms that guide glia and trachea distribution in the brain may explain, in part, shared gene regulatory pathways, including those regulated by the related PAS proteins, Sim and Trh. Most of the *Drosophila* PAS proteins that interact with Tgo are expressed in the trachea, including Trh, Dys and Similar (the fly version of HIF-1α), and Sim likely descended from a common ancestral gene. Developmental functions of Sim and Trh may have arisen later than their adult functions and common ancestral functions of these two tissues in the adult may explain why many enhancers drive expression in both the midline and trachea and why other midline and tracheal enhancers are closely linked. Further dissecting the similarities and differences in gene regulation within the CNS midline and trachea will reveal novel molecular mechanisms used to construct these tissues during development. Additional experiments are also needed to understand how signaling pathways combine with Sim and Trh to regulate genes in midline glia and trachea, not only in embryos, but also in larvae and adults, under different environmental conditions.

## Supporting Information

Table S1 **List of PCR primers used to generate fragments of the** ***CG33275*** **,** ***esg, liprin γ, Netrin, comm, moody*** **and** ***Ect3*** **genes that were tested for their ability to drive midline and tracheal transcription.** Restriction sites introduced for cloning purposes are indicated in lower case.
(DOC)

Table S2 **List of PCR primers used to generate the synthetic reporter genes tested for their ability to drive midline and tracheal transcription.** The ^1^ *Toll* and *Sox* synthetic reporters have been previously reported [59]. Engineered restriction sites used to ligate and subclone the synthetics are shown in lower case.
(DOC)

Table S3 **For each enhancer, ^1^the name of the enhancer, ^2^the tissue that expressed** ***GFP*** **driven by the enhancer, and ^3,4^PCR primers used to generate the enhancers derived from** ***esg*** **and** ***Netrin*** **genes are listed.** Restriction sites introduced for cloning purposes are indicated in lower case.
(DOC)

Table S4 **Listed is the immediate context of CMEs found within enhancers that drive expression in ^1^the midline, ^2^the trachea or ^3^both tissues.** For each CME (ACGTG), ^4^the gene where it is found, ^5^the number of CMEs within each enhancer and the ^6^seven bp sequence of the site are shown. ^7^The total number of CMEs found within the enhancers examined is indicated at the bottom of the table.
(DOC)

Table S5 **Listed is the immediate context of GCGTG motifs found within identified enhancers that drive expression in ^1^the midline, ^2^the trachea or ^3^both tissues.** For each GCGTG motif, ^4^the gene where it is found, ^5^the number found within each enhancer and the ^6^seven bp sequence of the site are shown. ^7^The total number of CMEs found within the enhancers examined is indicated at the bottom of the table.
(DOC)
