# Supplementary material for: A Comparison of Midline and Tracheal Gene Regulation during Drosophila Development
Source: PLoS One. 2014 Jan 20;9(1):e85518. doi: 10.1371/journal.pone.0085518 (PMC3896416; doi:10.1371/journal.pone.0085518)
Supplement: Table S1 — List of PCR primers used to generate fragments of the CG33275 , esg, liprin γ, Netrin, comm, moody and Ect3 genes that were tested for their ability to drive midline and tracheal transcription. Restriction sites introduced for cloning purposes are indicated in lower case. (DOC) [file pone.0085518.s001.doc]

**Table S1. Primers used to amplify enhancers.**

| **Reporter** | **Forward primer** | **Reverse primer** |
| --- | --- | --- |
| *CG33275 ML 2544* | AAAggtaccAGAAAAGCGACTCGAAGACGA | AAAtctagaTCATTGGCAAGTCCAGTCACA |
| *CG33275 ML 753* | TTGCGTCGAGTTGGCATT | AAAggtaccGCCGATCATGTTGTGTTC |
| *CG33275 TR 2507* | AAAtctagaTGTGACTGGACTTGCCAATGA | AAActcgagTGAGCACAGCACGAAGTCAAT |
| *CG33275 TR 1228* | AAAtctagaTGTGACTGGACTTGCCAATGA | TGAGTGTTTCTGTGAGCG |
| *CG33275 TR 1297* | CGCTCACAGAAACACTCA | AAActcgagTGAGCACAGCACGAAGTCAAT |
| *CG33275 TR 265* | AAAggtaccCACCTTCGTGATTTGTGGCTT | AAActcgagTGAGCACAGCACGAAGTCAAT |
| *esg TR C1* | ATAGCTGAGATCCCAGCCATGTTCC | TCA CTT ACT GCT CTG GAT CGA TCG TC |
| *esg ML C2* | GTGATATCCCTCCACTTGGTGTCACTG | TCC GAG AGG CTG AAA CTT CTC ACA |
| *esg TR C7* | GACCCTCTTTGGCAGCTTCAAACAC | TCG GAA ACT AGC CTC GTT GAG ATA GC |
| *liprinγ 3141* | TAGactagtGAATTGCTCCAATGCTGATGG | AATgctagcGCGAAGCGTCAAGAGAAATCA |
| *liprinγ 889* | AAAggatccAGTACACTGGGGAAA | AATgctagcGCGAAGCGTCAAGAGAAATCA |
| *liprinγ 600* | AAAggatccAGTACACTGGGGAAA | AAAggtaccGGCTGGCACTTGAATCGAA |
| *liprinγ 308* | AAActcgagTTCGATTCAAGTGCCAGCC | AATgctagcGCGAAGCGTCAAGAGAAATCA |
| *liprinγ 165* | AAAggtaccTGAGCCCGGGTTTATCATTCT | AAAggtaccGCGAAGCGTCAAGAGAAA |
| *Netrin 797* | AAAggtaccCGCGAAGATCAACAGGAT | AAActcgagGATGCAGCATCCAGCAAC |
| *comm dwn 2575* | AAAggtaccGTTGGGCTGAGCCATTAA | AAAggatccTCTCCCTCCCTCTAAGAA |
| *comm dwn 693* | AAAggatccGGGAAAAAGCGGGGATATCA | AAAggatccTCTCCCTCCCTCTAAGAA |
| *comm dwn 737* | AAAggatccCCTGCTTTTGCCCAGTTT | AAAtcgagTGATATCCCCGCTTTTTCCC |
| *comm dwn 446* | AAActcgagTGCACGCATTTCTGCTG | AAAggatccTCTCCCTCCCTCTAAGAA |
| *comm dwn 267* | AAAggatccGGGAAAAAGCGGGGATATCA | AAActcgagGTCAGCAGAAATGCAGTGCA |
| *moody 1970* | AAAccgcggAGGCTCAGGCTCTGGTAA | AAAggtaccGCAAGAGAGCAGAAGAGA |
| *moody 1221* | AAACccgcggAGGCTCAGGCTCTGGTAA | AAAccgcggGCGAGGTGAAAGAGTGAGTG |
| *moody 608* | AAAccgcggAGGCTCAGGCTCTGGTAA | AAAccgcggGCATTGATATGCCTCTGC |
| *Ect3 3194* | AAAggtaccCTGGAAAAGCGAAATTGGGG | AAAggatccCGCGATTAAGTGCAGAAC |
| *Ect3 1955* | AAAggtaccAAATAAACCCCTGGCCTG | AAAggatccCGCGATTAAGTGCAGAAC |
| *Ect3 2311* | AAAggtaccCTGGAAAAGCGAAATTGGGG | AAAggatccTAGACGTAGGACGACTTG |
| *Ect3 1071* | AAAggtaccAAATAAACCCCTGGCCTG | AAAggatccTAGACGTAGGACGACTTG |
| *Ect3 1456* | AAAggatccAAACTGCTCATGCCCACT | AAAggatccCGCGATTAAGTGCAGAAC |
| *Ect3 517* | AAAggtaccAAATAAACCCCTGGCCTG | AAAggatccAAACTGCTCATGCCCACT |
| *Ect3 572* | AAAggtaccAGTGGGCATGAGCAGTTT | AAAggatccTAGACGTAGGACGACTTG |
