# Supplementary material for: A Comparison of Midline and Tracheal Gene Regulation during Drosophila Development
Source: PLoS One. 2014 Jan 20;9(1):e85518. doi: 10.1371/journal.pone.0085518 (PMC3896416; doi:10.1371/journal.pone.0085518)
Supplement: Table S2 — List of PCR primers used to generate the synthetic reporter genes tested for their ability to drive midline and tracheal transcription. The 1 Toll and Sox synthetic reporters have been previously reported [59]. Engineered restriction sites used to ligate and subclone the synthetics are shown in lower case. (DOC) [file pone.0085518.s002.doc]

**Table S2. Primers used to generate synthetic reporters.**

| **Reporter** | **Forward primer** | **Reverse primer** |
| --- | --- | --- |
| ^1^*Toll* | aattcAAATTTGTACGTGCCACAGAg | aattcTCTGTGGCACGTACAAATTTg |
| ^1^*Sox* | aattcCACAATGACGTGCCACAGAg | aattcTCTGTGGCACGTCATTGTGg |
| *synth 1* | aattcTCGGAATATGCAACAGCAGCACGTTCTCTGAAGCGGAGAGTGTAATg | aattcATTACACTCTCCGCTTCAGAGAACGTGCTGCTGTTGCATATTCCGAg |
| *synth 2* | aattcACAATGACATTTTATCTCGGAATATGCAACAGCAGCACGTTCTCTGAAg | aattcTTCAGAGAACGTGCTGCTGTTGCATATTCCGAGATAAAATGTCATTGTg |
| *synth 3* | aattcTCGGAATATGCAACAGCAGCACGTTCTCTGAAGCGGAGAGTGTCGCg | aattcGCGACACTCTCCGCTTCAGAGAACG TGCTGCTGTTGCATATTCCGAg |
| *synth 4* | aattcTTTTAAGCAGCACGTTCTCTGAAGC GGAGAGTGTAATg | aattcATTACACTCTCCGCTTCAGAGAACGTGCTGCTTAAAAg |
| *synth 5* | aattcTGGTCTGTGGCACGTCTGAATTTCTGCTAAATGTCATTGTGCATA | aattcTATGCACAATGACATTTAGCAGAAATTCAGACGT GCCACAGACCAg |
| *synth 6* | aattcTCGGAATATGCGCCAGCAGCACGTTCTCTGAAGCGGAGAGTGTAATg | aattcATTACACTCTCCGCTTCAGAGAACGTGCTGCTGGCGCATATTCCGAg |
| *synth 7* | aattcTAATTATCTGTGGCACGTACAAATTTg | aattcAAATTTGTACGTGCCACAGATAATTAg |
| *synth 8* | aattcATGCAACTCTGT GGCACGTACAA ATTTg | aattcAAATTTGTACGTGCCACAGAGTTGCATg |
| *synth 9* | aattcTGTGTGAGAAACTTACTTTCAGCTCGGTTCCCACGCCTCTGTGGCACGTACAAATTTg | aattcAAATTTGTACGTGCCACAGAGGCGTGGGAACCGAGCTGAAAGTAAGTTTCTCACACAg |
| *synth 10* | aattcTGTGGCACGTCTGAAATGTCATTGTg | aattcACAATGACATTTCAGACGTGCCACAg |
| *synth 11* | aattcATTACACTCTCCGCTTCAGAGATAAAA AGCACGTACAAATTTg | aattcAAATTTGTACGTGCTTTTTATCTCTGAAGCG GAGAGTGTAATg |
| *synth 12* | aattcTGGTGGCCCATTACTTTCAGCTCGTGGGTGCATCCTCTGTGGCACGTACAAATTTg | aattcAAATTTGTACGTGCCACAGAGGATGCACCCACGAGCTGAAAGTAATGGGCCACCAg |
