# Supplementary material for: A Comparison of Midline and Tracheal Gene Regulation during Drosophila Development
Source: PLoS One. 2014 Jan 20;9(1):e85518. doi: 10.1371/journal.pone.0085518 (PMC3896416; doi:10.1371/journal.pone.0085518)
Supplement: Table S3 — For each enhancer, 1the name of the enhancer, 2the tissue that expressed GFP driven by the enhancer, and 3,4PCR primers used to generate the enhancers derived from esg and Netrin genes are listed. Restriction sites introduced for cloning purposes are indicated in lower case. (DOC) [file pone.0085518.s003.doc]

**Table S3. Additional *esg* and *Net* enhancers that drive expression outside the midline and trachea.**

| **^1^Reporter** | **^2^Tissue** | **^3^Forward Primer** | **^4^Reverse Primer** |
| --- | --- | --- | --- |
| ***esg G*** | no embryonic expression | ACAGCCCTTAACCAACGCCACC | TTCTACGACTGCGACACGCTGC |
| ***esgF*** | larval subesophageal ganglia, mouth | TCCACTCTCACTTGCTGGCG | ACTCGGCACTCGGCTCATTACA |
| ***esg E*** | hindgut, anterior ectodermal clumps | TTGGGACTCGATTTGAGCGCA | GCAAAAGGTGCATTGTCGGTTGAT |
| ***esg D*** | ectodermal stripes, posterior spiracles, mouth parts | TCGATGCTCGCCTCGAATTG | CGCAGGTTCAGTTGGGTTCACA |
| ***esg A*** | no embryonic expression | TCCACTCTCACTTGCTGGCG | ACTCGGCACTCGGCTCATTACA |
| ***esg C3*** | ectoderm, muscles, esophagus, posterior spiracles | TTCTGGTCCCAAATCGGAAGTGG | GTTTCTGATGTCTGGGTGTCTCCATTGC |
| ***esg C4*** | posterior spiracles, esophagus, ectoderm | AGGCAAACAAAGGAGACAAGAGAGTGG | TGCCGTGTCATAGTGGGAAAGTG |
| ***esg C5*** | ectoderm, esophagus, mouth parts | CGACTAGAGACAGGTTGGACATTCG | GTATATGGAGTGGTTCGGTGTGATCGG |
| ***esg C6*** | pharynx, esophagus, lateral CNS | TCTGCTGGCTTTCAGTTGGTGTCA | GACCATTAGCGTGTACTTGCAGCC |
| ***esg C8*** | mouth parts, anterior ectoderm head, posterior spiracles | AGTTGCGGTTTTGGTCAATGGCAAC | CCACCCTCAAGAGCCACAATGATCA |
| ***NetA 2380*** | somatic muscles | GATggtaccGCAAAGTCCAAACGGCTTT | CGAgcatgcGAACGACGGCAAAAAGGGAT |
| ***NetA 525*** | few cells near gut, ventral ectoderm | AAAggtaccCGGAGGAAGCCTACATAA | AAActcgagGTCTTCCTGCTCCTGTTT |
| ***NetA 2989*** | ectoderm, midgut | AAAccgcggTAGTGGCTTGCTTAACTCGC | TAAggtaccGCCCCATTCGAAAGCACAA |
| ***NetA 1090*** | muscles or ectoderm, larval mouth | AAAggtaccTAACGTCAAGTCGGGCTG | AAActcgagGCTCATGGTGATGAGGCA |
| ***NetA 2574*** | somatic muscles | TATggtaccCCTTTGGCCAGGCTAAACAA | GTAccgcggACATCAGTCGACGACTGC |
| ***NetA 2972*** | midgut | AAAtctagaTTGACGTTACGAACCGAACG | AATggtaccTAACGTCTCGATTTCGCCTC |
| ***NetB 4028*** | visceral muscles, dorsal vessel, pharynx, esophagus, gut | ATCctcgagCACGATCGCGATATTTTCCGG | AAAggtaccCTCCATATGGATTTGTGCGGC |
| ***NetB 2445*** | gut | ATCctcgagCATCGTCCTGATCCGAAAGTG | ATCactagtTTGTTTGAGAGTGCGAGAGTG |
| ***NetB 2787*** | lateral glia, posterior spiracle, muscles, mouth, esophagus | ATCactagtATCAGTTTCGACCCACTGTGC | ATCggtaccACGATGCAAGTGCAATGGTCA |
