# Supplementary material for: A Comparison of Midline and Tracheal Gene Regulation during Drosophila Development
Source: PLoS One. 2014 Jan 20;9(1):e85518. doi: 10.1371/journal.pone.0085518 (PMC3896416; doi:10.1371/journal.pone.0085518)
Supplement: Table S5 — Listed is the immediate context of GCGTG motifs found within identified enhancers that drive expression in 1the midline, 2the trachea or 3both tissues. For each GCGTG motif, 4the gene where it is found, 5the number found within each enhancer and the 6seven bp sequence of the site are shown. 7The total number of CMEs found within the enhancers examined is indicated at the bottom of the table. (DOC) [file pone.0085518.s005.doc]

**Table S5. Flanking context of GCGTG motifs within midline and tracheal enhancers.**

| **^1^Midline only** |  |  | **^2^Trachea only** |  |  | **^3^Midline and Trachea** | |  |
| --- | --- | --- | --- | --- | --- | --- | --- | --- |
| **^4^Gene** | **^5^Number** | **^6^Sequence** | **^4^Gene** | **^5^Number** | **^6^Sequence** | **^4^Gene** | **^5^Number** | **^6^Sequence** |
| *wrapper* | 1 | tgcgtgt | *moody* | 2 | tgcgtga | *link* | 1 | tgcgtga |
| *sim 1.0* | 3 | tgcgtgc |  |  | ggcgtgg | *Net* | 1 | agcgtgg |
|  |  | tgcgtgc | *CG33275TR* | 3 | cgcgtgt | *rho* | 2 | tgcgtgt |
|  |  | tgcgtgt |  |  | cgcgtgt |  |  | tgcgtgc |
| *glec* | 2 | agcgtgt |  |  | agcgtgt | *btl* | 0 |  |
|  |  | agcgtgg | *trh45* | 0 |  | *vvl* | 4 | ggcgtgg |
| *oatp26f* | 3 | ggcgtga | *trh47* | 0 |  |  |  | ggcgtgg |
|  |  | cgcgtga | *trh66* | 1 | tgcgtgc |  |  | cgcgtgt |
|  |  | tgcgtgc | *trh67* | 4 | agcgtgg |  |  | tgcgtgc |
| *CG33275ML* | 0 |  |  |  | cgcgtgc | *liprin γ* | 0 |  |
| *esg C2* | 3 | tgcgtga |  |  | tgcgtgc |  |  |  |
|  |  | tgcgtgc |  |  | agcgtga |  |  |  |
|  |  | tgcgtga | *esg C1* | 1 | tgcgtgc |  |  |  |
| *commdwn* | 0 |  | *CG15252* | 0 |  |  |  |  |
| *ect3* | 4 | tgcgtgt | *CG13196* | 1 | ggcgtga |  |  |  |
|  |  | ggcgtgc | *dys* | 1 | cgcgtgg |  |  |  |
|  |  | tgcgtgt | *vvl1+2* | 0 |  |  |  |  |
|  |  | tgcgtgt | *vvl345* | 1 | cgcgtga |  |  |  |
| *Toll* | 2 | tgcgtgt | *esg C7* | 4 | agcgtgt |  |  |  |
|  |  | tgcgtgg |  |  | agcgtgc |  |  |  |
| *slit* | 0 |  |  |  | tgcgtgg |  |  |  |
| *sim 2.8* | 2 | agcgtga |  |  | tgcgtga |  |  |  |
|  |  | tgcgtgg |  |  |  |  |  |  |
| *rst* | 2 | ggcgtgg |  |  |  |  |  |  |
|  |  | ggcgtgc |  |  |  |  |  |  |
| **^7^Total** | **22** |  |  | **18** |  |  | **8** |  |
